# *PVT1* Long Non-coding RNA in Gastrointestinal Cancer

**DOI:** 10.3389/fonc.2020.00038

**Published:** 2020-01-31

**Authors:** Águeda Martínez-Barriocanal, Diego Arango, Higinio Dopeso

**Affiliations:** ^1^Group of Biomedical Research in Digestive Tract Tumors, CIBBIM-Nanomedicine, Vall d'Hebron University Hospital, Research Institute (VHIR), Universitat Autònoma de Barcelona, Barcelona, Spain; ^2^Group of Molecular Oncology, IRB Lleida, Lleida, Spain

**Keywords:** *PVT1*, lncRNA, siRNA, ceRNA, Myc, colorectal/gastric cancer

## Abstract

Whole genome and transcriptome sequencing technologies have led to the identification of many long non-coding RNAs (lncRNAs) and stimulated the research of their role in health and disease. LncRNAs participate in the regulation of critical signaling pathways including cell growth, motility, apoptosis, and differentiation; and their expression has been found dysregulated in human tumors. Thus, lncRNAs have emerged as new players in the initiation, maintenance and progression of tumorigenesis. *PVT1* (plasmacytoma variant translocation 1) lncRNA is located on chromosomal 8q24.21, a large locus frequently amplified in human cancers and predictive of increased cancer risk in genome-wide association studies (GWAS). Combined, colorectal and gastric adenocarcinomas are the most frequent tumor malignancies and also the leading cause of cancer-related deaths worldwide. *PVT1* expression is elevated in gastrointestinal tumors and correlates with poor patient prognosis. In this review, we discuss the mechanisms of action underlying *PVT1* oncogenic role in colorectal and gastric cancer such as *MYC* upregulation, miRNA production, competitive endogenous RNA (ceRNA) function, protein stabilization, and epigenetic regulation. We also illustrate the potential role of *PVT1* as prognostic biomarker and its relationship with resistance to current chemotherapeutic treatments.

## Introduction

According to the latest data released by the International Agency for Research on Cancer (IARC), cancer of the digestive tract is the leading cause of cancer and cancer-related death worldwide (1–3) (Table 1). Digestive tract tumors include malignancies arising in the oral cavity, esophagus, stomach, small, and large intestines, rectum and anus. Briefly, oral cancer is a subgroup of head and neck cancers, characterized by the growth of tumor cells in the lining of the lips, mouth, and upper throat. Oral squamous cell carcinoma represents the most frequent oral neoplasm and arises from epithelial cells. The greatest risk factor for oral cancer is the tobacco and/or alcohol use. However, the exposure to chemical carcinogens, ultraviolet/ionizing radiation and viral infections, such as *Human Papilloma virus (HPV), Epstein-Barr Virus (EBV)* or *Hepatitis C virus (HCV)*, are also known to increase oral cancer incidence rate (4). Esophageal cancer occurs when malignant cells arise in the tissue of the esophagus, the tube that transports the food from the mouth to the stomach. There are two major subtypes of esophageal cancer, which are epidemiologically and biologically very distinct. Esophageal squamous cell carcinomas occur in the epithelial cells of the mucosa, are frequently found in the upper and middle sections of the esophagus and associate with risk factors leading to recurrent chemical or physical insults to mucosa, i.e., tobacco, alcohol, or hot drinks use. Conversely, esophageal adenocarcinomas arise from the glandular cells in the mucosa, are more abundant in the lower section of the esophagus, and are associated with gastro-esophageal reflux and obesity as the main risk factors (5, 6). In turn, gastric cancer occurs when malignant cancer cells grow and colonize the wall of the stomach. The most common form of stomach cancer is the gastric adenocarcinoma, which originates in the epithelial cells from the mucosa. Other less common types of stomach cancer include gastro-intestinal stromal tumors, which develop in the connective tissue from the stomach wall, squamous cell carcinomas and carcinoid tumors. Traditionally, gastric adenocarcinomas are divided into two main histological subtypes, namely intestinal and diffuse. These subtypes follow very different oncogenic programs and thus display different molecular profiles (7). The etiology of gastric cancer is multifactorial but dietary factors, such as high salt and nitrate intake, and *Helicobacter pylori* infection increase the risk of gastric cancer development and progression (8–10). And finally, colorectal cancer occurs when tumor cells grow in the colon or rectum inner lining. The normal intestinal epithelium is maintained by a tight balance of proliferation, migration and cell death. Tumorigenesis occurs when these mechanisms become deregulated resulting in cell hyperproliferation and loss of differentiation, evidenced by the formation of aberrant crypts evolving into adenomatous polyps and subsequently into adenocarcinomas. Only a small proportion of colorectal tumors display a mesenchymal origin. Alcohol consumption, smoking, high fat diet as well as obesity are well-known risk factors for colorectal cancer (11).

**Table 1:** Incidence and mortality rates of tumor malignancies in the digestive tract worldwide.

	**Incidence**				**Mortality**			
	**Number**	**Ranking**			**Number**	**Ranking**		
		**Males**	**Females**	**Both sexes**		**Males**	**Females**	**Both sexes**
Colorectal	1,849,518	3	2	3	880,792	4	3	2
Stomach	1,033,701	4	7	5	782,685	3	5	3
Esophagus	572,034	7	13	7	508,585	6	9	6
Lips/oral cavity	354,864	11	19	16	177,384	12	16	15
Total digestive tract	3,810,117			1 (21.1%)	2,349,446			1 (24.6%)

*Incidence and mortality numbers for digestive tract malignancies and position occupied in the ranking when compared to other cancers are shown. Percentages considering all tumor sites are indicated in brackets. Data was extracted from the 2018 Global Cancer Observatory (International Agency for Research on Cancer) (1)*.

Among all tumor types in the digestive tract, colorectal, and gastric cancers exhibit the highest incidence and mortality rates. Specifically, colorectal cancer is the third most common cancer type in men after lung and prostate cancer; and the second most common in women after breast cancer. Stomach cancer, in turn, ranks fourth and seventh regarding its incidence in men and women, respectively. Only in 2018, 1.8 million colorectal and about 1 million stomach cancer cases were diagnosed worldwide, accounting for almost 900,000 and 800,000 deaths, respectively (Table 1). Moreover, it is expected that by 2040, the incidence of colorectal cancer will raise by 30% and mortality by 40%; whereas statistics for stomach cancer will both worriedly increase by 60% due to its high prevalence in Asian countries, whose population and economic growth rates are increasing. The prognosis of gastrointestinal cancer patients largely depends on tumor stage at the time of diagnosis (Table 2). Additionally, tumor staging strongly influences the clinical management (12–15). Thus, gastrointestinal cancer represents a major health and social issue requiring great investment from governments worldwide to cover the cost of prevention, diagnosis and treatment.

**Table 2:** Survival rates for colorectal and gastric cancer.

**Stage**	**Colorectal**		**Stage**	**Stomach**
	**Colon**	**Rectum**		
0	–	–	0	–
I	92	87	IA	92
IIA	87	80	IB	87
IIB	63	49	II	63
IIIA	89	84	IIIA	89
IIIB	69	71	IIIB	69
IIIC	53	58		
IV	11	12	IV	53

*Five-year survival rates (%) for colorectal and gastric cancer patients are shown according to the tumor staging system. Stage 0: Abnormal cells are found but confined in the tissue of origin; Stage I: Tumor cells are found but confined in the tissue of origin; Stage II: Tumor cells have spread deeper into the gastrointestinal wall; Stage III: Tumor cells have spread into nearby lymph nodes; Stage IV: Tumor cells have spread to distant tissues and organs. Data was extracted from ESMO Clinical Practice Guidelines (European Society for Medical Oncology) (12–15)*.

Efficient clinical management of cancer largely relies on the identification and study of key mediators of the tumorigenic process, both at onset and progression. Classically, most of the research efforts in tumor biology have been focused on protein-coding oncogenes, tumor-suppressor genes and DNA repair genes. But with the irruption of whole-transcriptome sequencing (RNAseq) technologies and computational sciences, we have gained great insights into non-coding RNAs (ncRNAs). These RNAs cover over 90% of the human genome and regulate a great variety of cellular processes including chromatin architecture and remodeling, transcription, post-transcriptional modification, epigenetic regulation, and signal transduction, both in physiological and pathological processes including cancer (16–18). An arbitrary 200-nucleotide length cut-off allows the classification of non-coding transcripts into two categories: short and long ncRNAs. Short ncRNAs are represented by microRNAs (miRNAs), PIWI-interacting RNAs (piRNAs), transcription initiation RNAs (tiRNAs), small nucleolar RNAs (snoRNAs), promoter-associated small RNAs (PASRs), promoter upstream transcripts (PROMPTs), and TSS (transcriptional start site)-associated RNAs (TSSa-RNAs). On the other hand, long ncRNAs (lncRNAs) group heterogeneous non-coding transcripts such as intergenic non-coding RNAs (lincRNAs), ultraconserved regions (T-UCRs) and other ncRNAs collectively named lncRNAs (17, 19). miRNAs have been the most widely studied class of ncRNAs in tumor biology. Since its discovery two and a half decades ago, many miRNAs have been implicated in the development of multiple human cancers through a wide range of mechanisms. Among all these mechanism, gene silencing has been predominant (20, 21). Mirroring what we have learned about cancer-associated coding genes, we can classify miRNAs into oncogenic or tumor suppressor (22). Moreover, miRNAs have become actionable targets for cancer treatment and several therapeutic agents are under development including some that have reached clinical trials (23–25). Currently, there is a growing interest in lncRNAs biology and their role in the tumorigenic process. These transcripts are generated by RNA polymerase II, can be capped, spliced and polyadenylated, but lack an obvious open reading frame. According to the latest release of the GENCODE project (GRCh38.p12), which aims to build an encyclopedia of genes and gene variants, the human genome contains 16,066 lncRNA genes encoding for 29,566 different lncRNA transcripts (26–29). This number represents 27% of all annotated human genes.

Next-generation sequencing of large numbers of tumor specimens has revealed thousands of lncRNAs aberrantly expressed in a broad spectrum of cancers (30, 31). Dysregulation of certain lncRNAs leads to the hyper- or hypoactivation of cellular pathways that promote and/or sustain tumor initiation and progression (32). LncRNAs are involved in a broad range of processes such as transcriptional regulation of neighboring protein-coding genes, interference of miRNAs via sequence complementary, protein decoy, protein stability control, post-transcriptional processing, epigenetic regulation, high-order chromosomal dynamics, telomere biology, and subcellular structural organization (32–35). LncRNAs mediate all these functions through the interaction with proteins, RNAs and lipids. It is remarkable that unlike other ncRNAs, the function of lncRNAs cannot be inferred from sequence or structure and thus, experimental evaluation needs to be conducted for a full and accurate biological annotation (30). Because of their undeniable role in cancer biology, the therapeutic targeting of oncogenic lncRNAs and lncRNAs involved in resistance to treatment has raised significant attention. The main strategies to inactivate oncogenic lnRNAs aim their post-transcriptional degradation with siRNAs, or the steric blockade of lncRNA-protein interactions with small molecules, morpholinos, or antisense oligonucleotides (36, 37). Noteworthy, some intrinsic features of lncRNAs make them very attractive as cancer diagnostic and prognostic biomarkers (38). First, lncRNAs are expressed in a more tissue-specific manner than protein-coding genes. It has been estimated that 78% of all lncRNAs are tissue-specific, while protein-coding genes barely reach 19% of specificity (39). Additionally, lncRNAs dysregulation in primary tumors and metastasis is observed in body fluids, i.e., whole blood, plasma, urine, saliva, and gastric juice (40). And despite the high abundance of ribonucleases in most of these fluids, lncRNAs are easily detected due to its high stability. This represents a clear advantage to patients as it allows cancer diagnosis and follow-up using minimally invasive methodologies.

Cancer cells show a plethora of chromosomal abnormalities, including translocations, amplifications, and deletions. The plasmacytoma variant translocation 1 (*PVT1*) gene encodes a lncRNA that was first identified when studying a recurrent translocation breakpoint in the *Ig*κ locus found in murine plamacytomas (41). One year later, a homologous human sequence was identified when studying immunoglobulin translocations in Burkitt lymphoma (42). We now know that *PVT1* gene fusions occur in additional hematologic malignancies, such as non-Hodgkin lymphoma and advanced multiple myeloma (10, 43, 44). *PVT1* gene fusions are also found in solid tumors although at much lower rates (10, 43, 44). *PVT1* exon 1 and intron 1 are most often involved in these DNA rearrangements (45). In addition, human *PVT1* is a target of genetic gains and amplifications in a large variety of cancers, including those of the digestive tract (46, 47). Moreover, genome-wide association studies (GWAS) identified single nucleotide polymorphisms (SNPs) in the *PVT1* locus (8q24) that are associated with increased colorectal cancer risk (48). Of interest, *PVT1* locus leads to the production of a cluster of four annotated miRNAs, namely miR-1204, miR-1205, miR-1206, and miR-1207 (-5p and -3p), being some of them important in the tumorigenic process of colorectal and gastric cancer (49–52). It is of note that although *PVT1* has been mostly studied in the context of cancer, this lncRNA is related to multiple and diverse pathologies (Table 3).

**Table 3:** *PVT1* in disease.

**Disease**	**Reports**
Cancer	335
Diabetic nephropathy	9
Arthritis	4
Cardiac disease	3
Diabetic neuropathy	2
Asthma	2
Vascular disease	1
Chronic obstructive pulmonary disease	1
Epilepsy	1
Schizophrenia	1
Multiple sclerosis	1
Muscle atrophy	1
Diabetic retinopathy	1
Sepsis	1
Immunodeficiencies	1
Vitiligo	1
Pulpitis	1

*Human pathologies in which PVT1 has been involved. Number of reports in Pubmed database are shown*.

In this review, we will discuss the current knowledge of *PVT1* alteration/dysregulation, as well as its contribution to gastrointestinal cancer.

## *PVT1* Expression in Normal and Tumor Tissue

**Figure 1:**
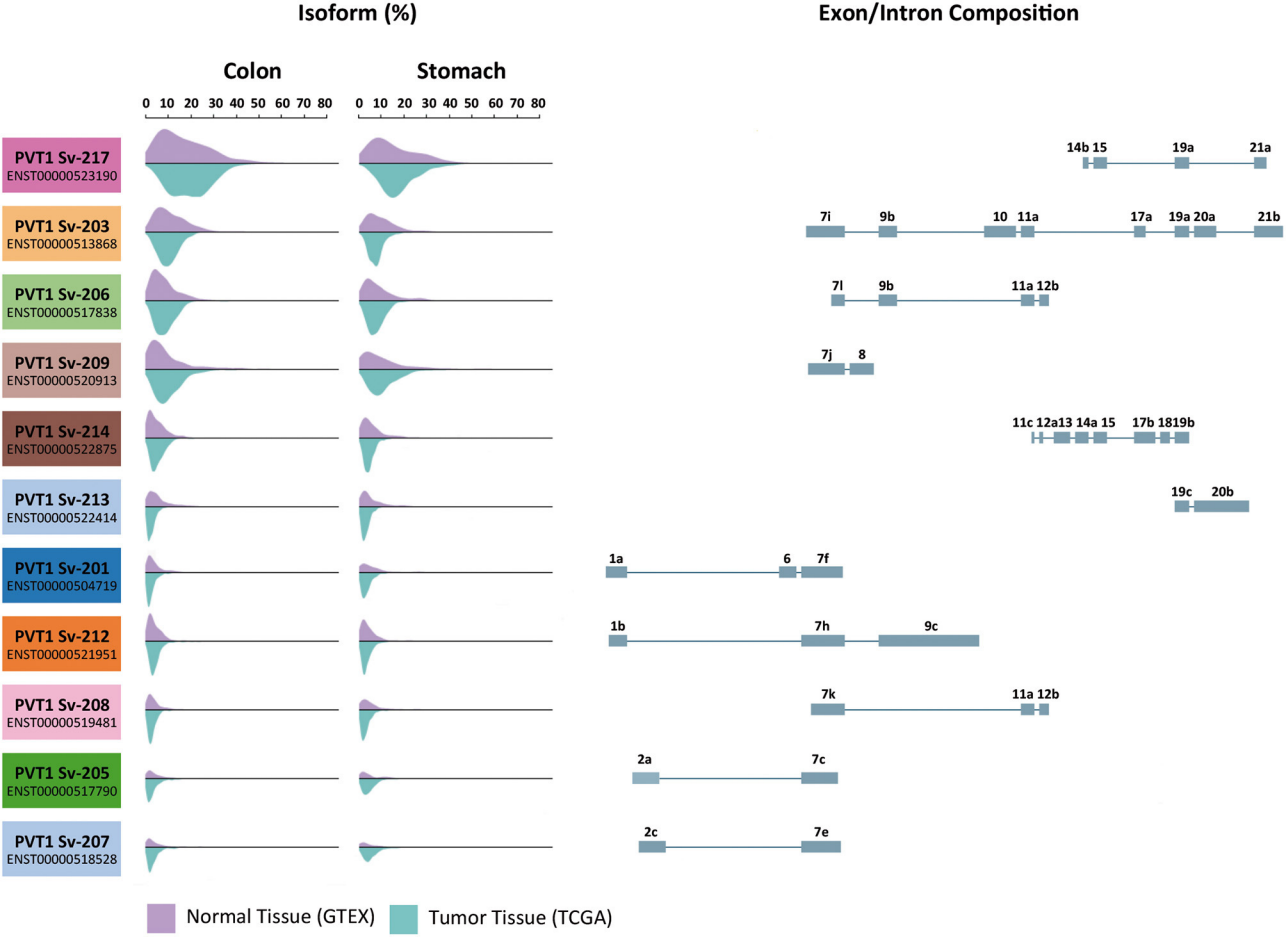
Expression of *PVT1* isoforms in normal and tumor tissue from the gastrointestinal tract. Eleven of the twenty-five predicted *PVT1* isoforms are expressed in the gastrointestinal tract. Transcripts variants are ranked from top to bottom according to their expression abundance (%) both in colon and stomach. Purple histograms illustrate the expression in normal tissue samples from GTEX database (colon *n* = 204 and stomach *n* = 204). Blue histograms illustrate the expression in tumor tissue samples from TCGA repository (colon adenocarcinoma *n* = 723 and stomach adenocarcinoma *n* = 453). Exon display for each *PVT1* transcript variant is shown. Data was extracted using UCSC Xena Browser.

Most lncRNAs exhibit a great number of isoforms and *PVT1* is not an exception (53). Human *PVT1* locus resides in chromosome 8q24.21 and contains 21 exons leading to 25 annotated transcript variants (54). These variants arise as a consequence of alternative splicing mechanisms mediating exon skipping and the use of unconventional donor and acceptor splice sites. Thanks to consortia such as The Genotype-Tissue Expression Project (GTEx) and The Cancer Genome Atlas (TCGA), we know that among all these *PVT1* transcripts, 14 are present in tissues at detectable levels (7, 55–58). Specifically, 11 *PVT1* transcripts have been detected in the normal gastrointestinal mucosa and adenocarcinomas of the colon and stomach (Figure 1). Among them, *PVT1*-217 (ENST00000523190) containing only four exons, is the most abundant *PVT1* transcript in the digestive tract. This heterogeneity in isoform expression needs to be considered when studying the role of *PVT1* in carcinogenesis, as the biology of each isoform might have a different impact on tumor initiation/progression and patient survival. Several oncogenic mechanisms have been attributed to *PVT1*, i.e., as a ceRNA (competing endogenous RNA) for several miRNAs and as a source of miRNAs itself (49, 51, 59–65). As a non-coding gene, all of them rely into *PVT1* RNA primary and secondary structure. Accordingly, the biological activity of *PVT1* depends on the sequence of the specific *PVT1* transcripts expressed in a given tissue at a given time. Unfortunately, very few reports have taken into consideration the large heterogeneity in *PVT1* isoform expression and consequently, some of the results described in this review require further investigation. Nonetheless, multiple studies assessing *PVT1* expression by means of microarray technology (66–68) or RT-PCR using oligonucleotides amplifying several *PVT1* isoforms, have shown a general overexpression of *PVT1* in colorectal tumors compared to paired normal tissue samples (66, 68–74). Only He et al. have examined the expression of individual *PVT1* splice variants (Sv) in colorectal cancer (68). They found *PVT1* Sv-214, Sv-205, Sv-209, Sv-208, Sv-206, Sv-207, Sv-213, Sv-219, Sv-201, and Sv-215 upregulated in colorectal cancer vs. normal samples (variants ranked by decreasing overexpression fold) (68). Similarly, increased *PVT1* expression has been described in primary gastric tumors compared to the normal gastric mucosa (75–78). *PVT1* isoform Sv-214 overexpression was found in primary gastric tumor samples compared to adjacent normal gastric tissue (79). In good agreement, increased *PVT1* expression in COAD (colorectal adenocarcinoma), READ (rectal adenocarcinoma) and STAD (stomach adenocarcinoma) samples compared to the corresponding normal tissue was revealed using transcriptomic expression data available at the TCGA repository (57, 80). Interestingly, the tumorigenic process does not seem to affect the relative abundance of *PVT1* isoforms (Figure 1), being *PVT1* Sv-217 the isoform also more abundant in tumors. Unfortunately, the studies investigating the expression and role of *PVT1* in gastrointestinal malignancies have not addressed directly or indirectly the expression of this isoform (66, 68–72, 75–78). All these observations have been extended to human cancer cell lines. Precisely, CACO2, SW480, SW620, HT29, and HCT116 cells derived from human colorectal tumors displayed higher levels of total *PVT1* compared to NCM460, FHC and HCoEpiC normal colonic epithelial cells (67, 70, 72, 81). Likewise, AGS, MKN45, SGC7901, and BGC823 gastric cancer cell lines have higher *PVT1* expression compared to GES1 normal gastric epithelium cell line (76, 79).

**Figure 2:**
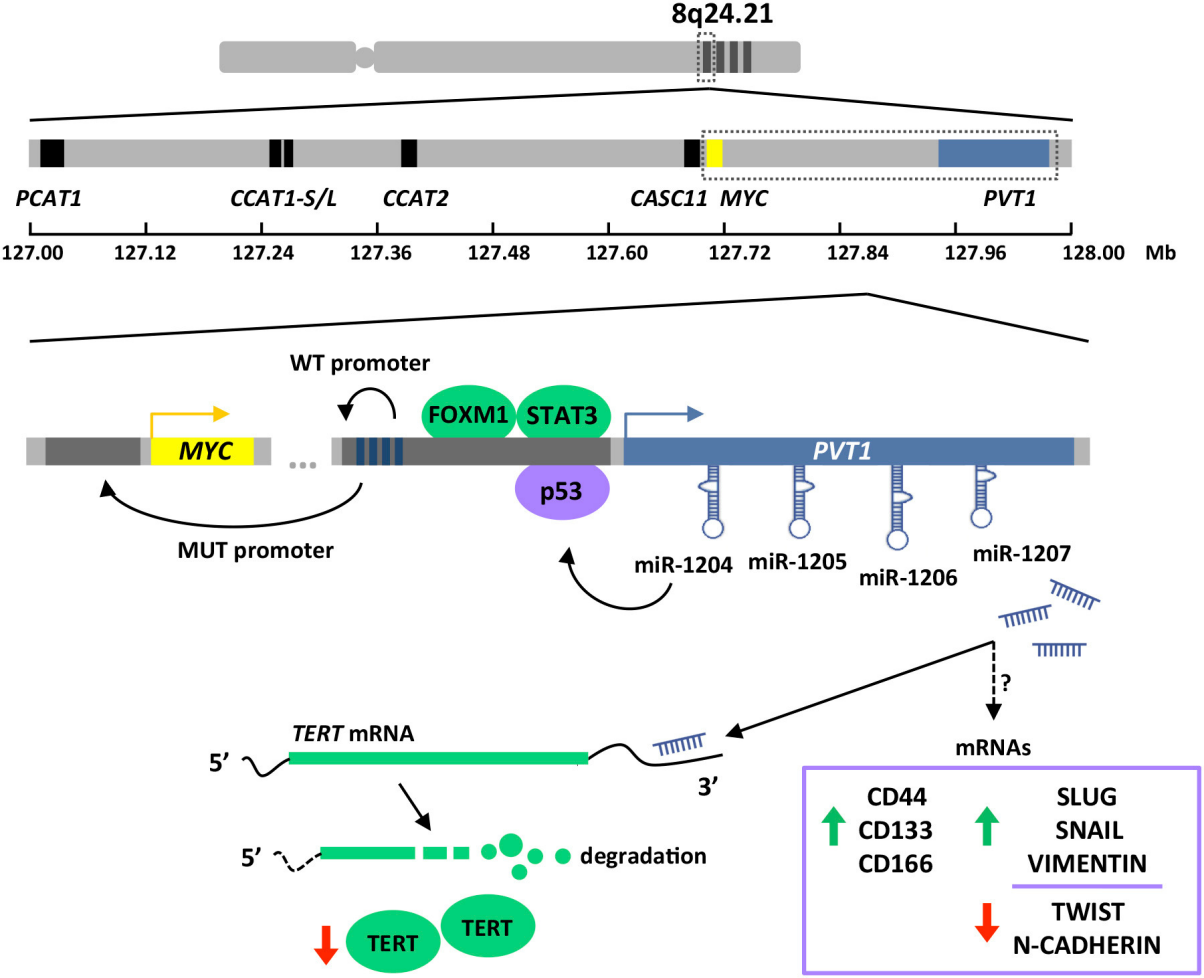
Chromosome 8q24.21 amplification and *PVT1* gene structure and regulation. 8q24.21 chromosomal region is frequently amplified in colorectal and gastric cancers (upper). This region contains *MYC* as the only protein-coding gene (yellow boxes) and several lncRNAs. Those lncRNA involved in *MYC* or *WNT* regulation have been depicted (blue and black boxes) (middle). *PVT1* lncRNA expression is regulated by FOXM1, STAT3, and p53 transcription factors. *PVT1* promoter exhibits four enhancer regions that in normal conditions (WT) boost its own expression. Due to *PVT1* promoter mutations and rearrangements (MUT), which are frequently found in cancers, enhancer elements preferentially interact with *MYC* promoter enhancing the transcription of the oncogene. *PVT1* encodes for four miRNAs. miR-1204 has been show to stabilize p53 protein, while miR-1207 has been proven to modulate TERT protein at the post-transcriptional level and expression of stemness and EMT genes by mechanisms currently unknown (lower). Pictograms in purple and green illustrate evidences obtained in colorectal and gastric cancer, respectively. Dark gray boxes indicate gene promoters. Dark blue boxes indicate enhancer regions. Green and red arrows indicate up- and down-regulation, respectively.

*PVT1* expression in stomach cancer is directly regulated by FOXM1 (Forkhead Box M1) transcription factor (75). FOXM1 controls the expression of genes such as *MYC, CCNB1, AURKB*, and *SKP2*, which are essential for cell cycle progression at DNA replication and mitosis, and therefore important for tumor initiation and progression (82–85). Additionally, FOXM1 plays a key role in DNA damage checkpoint participating in the repair of DNA strand breaks (86). *PVT1* transcript levels changes upon manipulation of FOXM1 protein expression in gastric cancer cells. Specifically, *PVT1* expression is reduced upon FOXM1 silencing and increases after FOXM1 overexpression (75). This regulation occurs through a direct interaction of FOXM1 with two independent binding sites in the *PVT1* promoter that enhances the transcriptional activation of this lncRNA (75). In addition, STAT3 (Signal Transducer and Activator of Transcription 3) has also been shown to regulate the expression of *PVT1* (77). Persistent STAT3 activation in tumor cells results in increased cell proliferation, survival, and invasion; and at the same time, STAT3 exerts non-cell autonomous effects in the tumor microenvironment by boosting tumor-promoting inflammation and suppressing anti-tumor immunity (87). STAT3 overexpression in gastric cancer cell lines leads to increased *PVT1* levels, while STAT3 knockdown results in *PVT1* transcriptional downregulation. These effects are explained by the presence of three canonical STAT3 binding motifs within the *PVT1* promoter, which were confirmed to control the transcription of this lncRNA (77) (Figure 2).

In colorectal cancer, *PVT1* transcription is influenced by p53 (50). This tumor suppressor is involved in the cell cycle regulation by transactivating a plethora of protein-coding but also non-protein-coding genes, that ultimately prevent cell division by inducing cell cycle arrest, senescence, or apoptosis (88, 89). Importantly, p53 is the most frequently mutated gene in human cancers (90, 91). The promoter of *PVT1* harbors a functional p53 response element that enhances the transcription of this lncRNA in colorectal cancer cell lines exposed to the DNA damaging agent daunorubicin, or upon increased p53 protein levels achieved by the use of Nutlin-3a (50). It is important to mention that *PVT1* upregulation upon these conditions was only monitored in p53 wild-type colorectal cancer cell lines and thus, it remains unknown the ability of mutant p53 proteins to exert an equivalent role. Although most p53 mutations result in expression of dominant-negative forms, certain mutations are known to confer oncogenic functions (92) (Figure 2).

## *PVT1* and MYC

**Figure 3:**
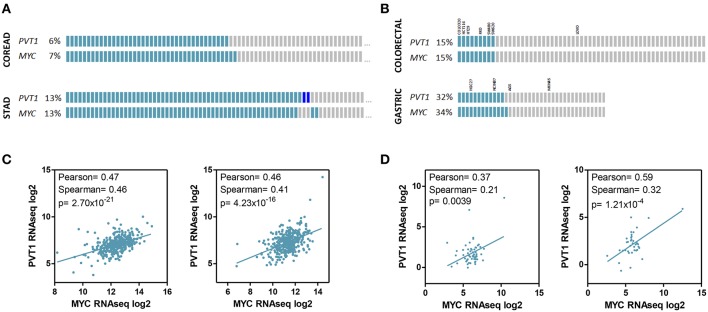
*PVT1* and *MYC* expression in gastrointestinal tumors and cell lines. *PVT1* and *MYC* copy number variation **(A,B)** and RNAseq expression **(C,D)** in tumor samples from colorectal (*n* = 616) and stomach *(n* = 441) adenocarcinomas **(A,C)** and established cell lines from colorectal (*n* = 58) and gastric tumors (*n* = 38) **(B,D)**. The name of cell lines used in studies investigating the role of *PVT1* in gastrointestinal cancer are shown. Gray boxes indicate no alteration. Light blue boxes indicate gene amplification. Dark blue boxes indicate gene deletion. RNAseq expression units: RPKM (Reads Per Kilobase Million). Pearson and Spearman correlation coefficients and associated *p*-value (Spearman) are shown.

High-resolution analyses of somatic copy-number alterations indicate that 8q24.21 region is one of the most frequently amplified regions across human cancers, including colorectal and gastric cancer, and are often associated with poor prognosis and/or drug resistance (7, 58, 93). Interestingly, although the amplified region spans almost 2 Mb the only protein coding gene in this locus is the *MYC* oncogene. This fact led to nickname this region as the gene “dessert” locus, and attribute to *MYC* the tumor promoting effects of gaining supernumerary copies of 8q24 (66, 94–96). Conversely, 8q24.21 has been shown to be an “oasis” for lncRNAs. Up to 12 lncRNAs have been identified in this locus, and very importantly for colorectal cancer, many of them regulate Wnt signaling pathway and in turn Myc activity (35, 69) (Figure 2). Wnt signaling is crucial for the initiation and maintenance of colorectal cancer and consistently, this pathway is altered in more than 90% of these malignancies according to the TCGA data (58). Wnt dysregulation in gastric tumorigenesis is less frequent but affects 10–30% tumors (7, 97). Accordingly, *PVT1* and *MYC* coamplification (66, 78, 98) and coexpression (71, 98, 99) has been observed in colorectal and gastric primary tumors and cell lines (Figure 3).

The first evidence of *PVT1* in human solid malignancies, and also its relationship with *MYC*, came from the study of COLO320, a colorectal cancer cell line that was known to be *MYC* amplified. Two variants of the cell line were isolated: COLO320-HSR (homogeneous stained region) with *MYC* allele intrachromosomically amplified; and COLO320-DM, with a rearranged and amplified *MYC* allele that is carried on small fragments of extrachromosomal DNA named double minutes (100). By studying *MYC* amplification in COLO320-DM, the authors noticed that intron 1 from *MYC* contained an ectopic DNA fragment which they characterized and described as *PVT1* in humans (101). However, the architecture of the *MYC*-*PVT1* locus in COLO320-DM was later realized to be unique, since *PTV1* locus normally lies 57 kb downstream of *MYC* gene. This chromosomal organization is conserved in other species such as mouse and rat, with syntenic 15qD1 and 7q33 regions, respectively (99).

The expression of *PVT1* and MYC in tumors are correlated not only because of their genetic coamplification. The 8q24 region displays a very strong enhancer activity creating feedback loops controlling MYC expression (102). Precisely, lncRNAs within 8q24 region are efficiently transcribed due to the presence of abundant transcriptional factors and mediator proteins, and these lncRNAs in turn, increase the enhancer activity by forming chromatin loops and protein bridges to promote the transcription of *MYC* (71, 98, 103). Interestingly, *PVT1* promoter is recurrently rearranged in human tumors, including those affecting the digestive tract (10, 44, 104). It has been demonstrated experimentally that insertions or deletions in the *PVT1* promoter lead to Myc overexpression and consequently, enhanced growth in cancer cells (104). Chromatin is organized into self-interacting units called topologically associating domains (TADs). Contacts between promoter and enhancer regions take place within the TADs, leading to an additional layer of gene expression regulation (105). As a consequence, genomic DNA structural variations, i.e., deletions, duplications, insertions, inversions, and translocations, are able to modify the three-dimensional chromatin topology and promote or suppress promoter-enhancer interactions within the TADs (104, 106). *PVT1* gene harbors four intragenic enhancer elements that under normal conditions establish strong interactions with the *PVT1* promoter and sustain expression of the non-coding transcript. However, under the *PVT1* promoter mutational rearrangements found in tumor cells, and even epigenetic inactivation, *PVT1* intragenic enhancer elements interact preferentially with *MYC* promoter, thus boosting the transcription of *MYC* oncogene and its oncogenic activity of tumor cells (104) (Figure 2).

## Oncogenic *PVT1* Functions in Colorectal and Gastric Cancer

LncRNAs carry out their biological functions through multiple and diverse mechanisms. Several research groups have demonstrated the overall ability of *PVT1* to sustain *in vitro* and *in vivo* cell growth, clonogenicity, migration, and invasion, both in colorectal (66–68, 71, 72, 107) and gastric cancer epithelial cells (75–77, 79). The capacity of *PVT1* to negatively regulate apoptosis through the inhibition of the TGFβ pathway, is more controversial, as it has not been observed systematically (66, 70–72, 78, 79, 107, 108). *PVT1* lncRNA has been shown to exert its protumorigenic activity also in a non-cell autonomous manner. Precisely, *PVT1* expression in gastric tumors enhances microvessel formation, both *in vitro* and *in vivo*, through a mechanism that involves vascular endothelial growth factor A (VEGFA) expression in a STAT3-dependent manner (77). Formation of new vessels is a crucial step for tumors to maintain the supply of oxygen and nutrients, and VEGFA is a master regulator of this process (109). Consistently, antiangiogenic agents are routinely used in the clinical practice to treat cancer patients (110, 111). Antiangiogenics are used in combination with chemotherapy in the first line of treatment of colorectal cancer, and several compounds targeting tumor vasculature have been recently approved for the management of gastric cancer (13, 14, 112). In fact, expression of *PVT1* and VEGFA in combination predicts poor survival in gastric cancer, further supporting the use of antiangiogenic drugs in these patients (77).

All these results have been obtained upon genetic manipulation of *PVT1* levels in cell line models, and it is important to mention that these manipulations have not been restricted to a single *PVT1* isoform (68). The siRNAs often used to achieve downregulation of *PVT1* target multiple transcripts, and not always those predominantly expressed in colorectal and gastric tumors. On the other hand, models of *PVT1* overexpression do not adequately report the specific isoforms used. The only exception is the study of He et al., where they convincingly demonstrated in HCT116 and SW480 colon cancer cell lines, the ability of *PVT1* Sv-214 to promote proliferation, stemness, migration and invasion *in vitro*; as well as *in vivo* by establishing tumor xenografts and liver metastasis mouse models in immunodeficient nude mice (68). Importantly, differences in the *PVT1* isoforms downregulated in different studies might explain the discrepancies reported regarding the role of *PVT1* to regulate apoptosis. In this line, the suitability of certain *PVT1* knockdown cellular models exhibiting apoptosis to assess invasion and/or migration properties should be reconsidered.

### miRNA Production

**Figure 4:**
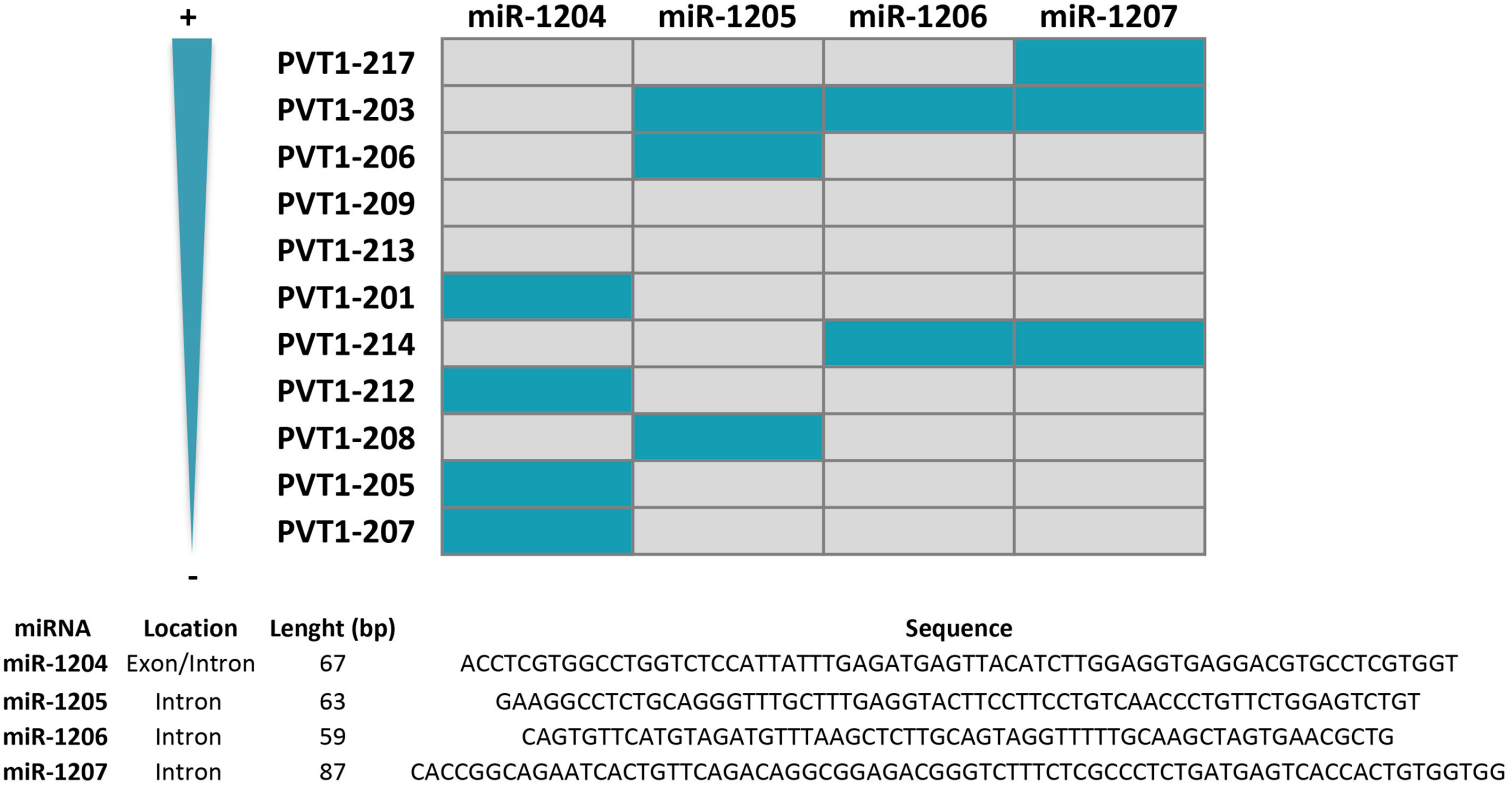
miRNAs encoded by *PVT1* transcript variants. *PTV1* isoforms are ranked according to expression abundance (%) in gastrointestinal normal tissues and tumors. Gray and blue boxes indicate absence and presence of the indicated miRNA, respectively. Nucleotide sequence, length, and location within *PVT1* introns or exons is indicated for all miRNAs.

*PVT1* locus leads to the production of a cluster of four annotated miRNAs, namely miR-1204, miR-1205, miR-1206, and miR-1207 (-5p and -3p) (49, 51) (Figure 2). Originally, miR-1208 was also mapped into *PVT1* locus, but according to latest version of Ensemble Genome browser (v98) this miRNA lies outside the lncRNA sequence (54). All these miRNAs are conserved in the mouse *Pvt1* gene (51). As depicted in Figure 4, most of the miRNAs contained within the *PVT1* transcript variants expressed in the colon and stomach are found in intronic regions. Spatiotemporal organization of the RNA splicing has important implications to fully understand the *PVT1* loss-of-expression experiments found in the literature (113). Gene silencing using exogenous siRNA against exonic regions does not necessary entail that the miRNAs encoded within introns will also be silenced. Upon splicing in the nucleus, introns are available for microRNA processing while the spliced transcript is exported to the cytoplasm where it may be targeted by exogenous siRNAs. This might explain why the use of siRNAs against *PVT1* in HCT116 and RKO cells did not have any effect on the expression of miR-1204, miR-1205, and miR-1207-5p/3p (66). Contrary, expression of all *PVT1*-encoded miRNAs was enhanced upon exposure of the cells lines to the DNA damaging agent daunorubicin, or upon increased p53 protein levels achieved through Nutlin-3a stimulation (50). This, effect is likely to be indirect because, as mentioned before, *PVT1* increases as consequence of p53 stabilization. Among the four *PVT1*-encoded miRNAs, only miR-1204 seems to engage a positive feedback loop to sustain stabilization of p53 at protein level (50).

The role of *PVT1*-encoded miRNAs in the initiation and/or progression of malignancies in the stomach or colon is uncertain due to the lack of literature. Only miR-1207 is reported to have an effect in gastric cancer proliferation and invasion, or in the stemness properties of colon cancer cells (52, 81). Specifically, it has been shown how ectopic expression of miR-1207-5p in the SGC7901 gastric tumor cell line is able to reduce proliferation and invasion *in vitro* and *in vivo* by targeting the catalytic subunit of the telomerase complex hTERT (52). Aberrant expression of hTERT is associated with the metastatic ability of gastric tumors (114, 115). In turn, miR-1207-5p overexpression in the HCoEpiC normal colon epithelial cell line enables the formation of primary and secondary spheres by a mechanism involving the upregulation at the mRNA level of TGFβ, CTNNB1, MMP2 and several colorectal stem cell markers such as CD44, CD133, and CD166 (81). Consistent with the tight relationship between stemness and epithelial-mesenchymal transition (EMT), Slug, Snail, and vimentin were also found upregulated (81, 116) (Figure 2). Interestingly, miR-1207-5p miRNA seems to exhibit oncogenic activity in colorectal cancer while having tumor suppressor effects in gastric cancer cells (contrary to the oncogenic role of *PVT1*).

### ceRNA

LncRNAs have the ability to modulate gene expression indirectly by impairing miRNA activity through sequestration/sponging, which leads to an effective de-repression of targets of these miRNAs (33, 34). This function, named competitive endogenous RNA (ceRNA), has encountered certain skepticism due to the fact that the physiological expression levels of an individual lncRNA may not be sufficient to completely suppress the activity of miRNAs (117). However, regulation by modestly expressed lncRNAs could be magnified through downstream processes, particularly through the upregulation of transcription factors that by transactivating multiple targets contribute to outcome amplification. This might be the case for *PVT1*, which as shown here maintains a close relationship with Myc, p53 and STAT3, all of them key transcription factors in the tumorigenic process.

#### miR-152

**Figure 5:**
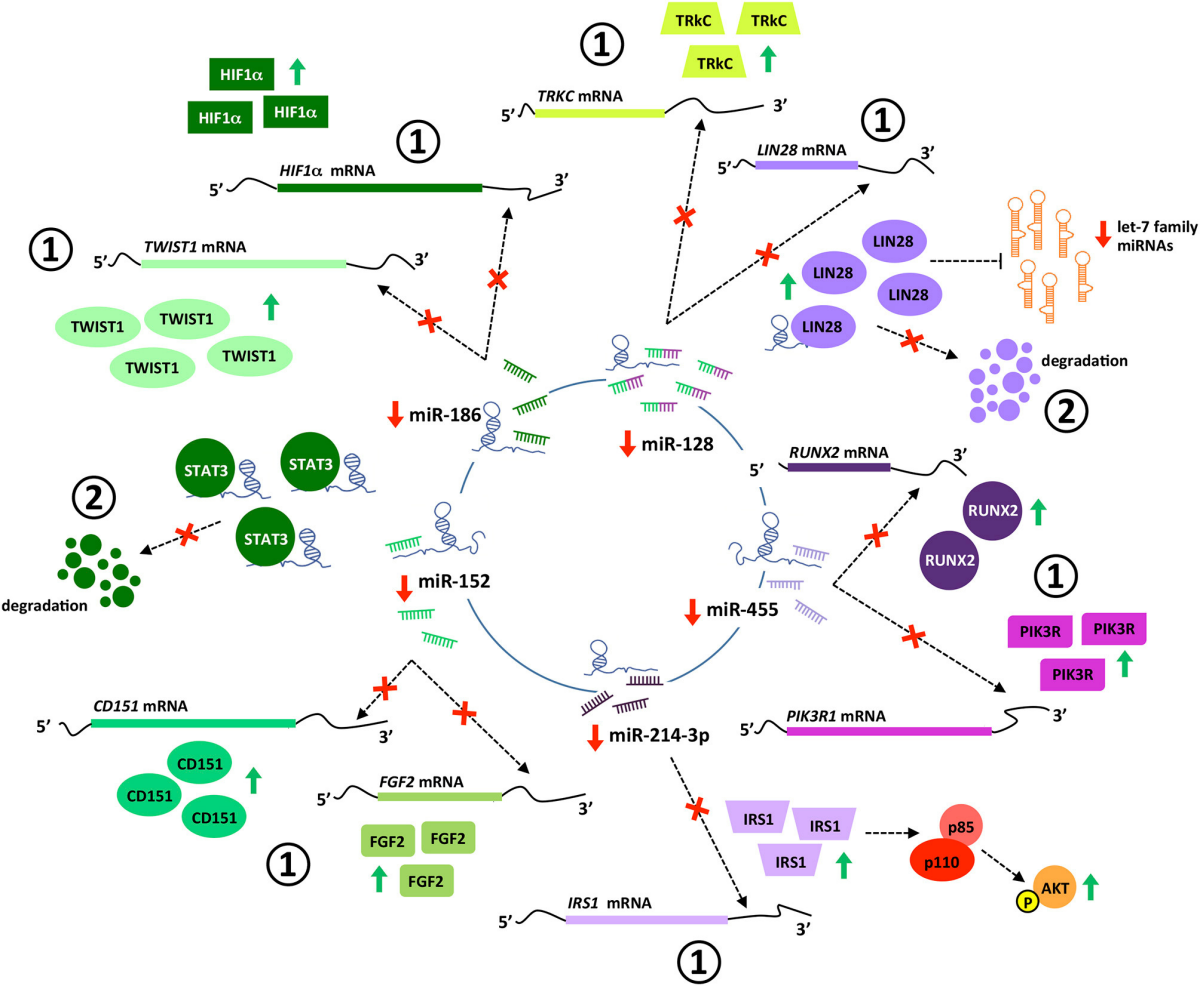
*PVT1* interaction with miRNAs and proteins. *PVT1* interacts with multiple miRNAs and proteins in cancer cells, leading to post-transcriptional (1) and post-translational (2) regulation of gene expression. *PVT1*-miRNA interactions promote efficient de-repression of miRNA targets. Specifically, *PVT1* hinders miRNAs binding in the 3′ untranslated region of target transcripts, preventing their degradation by the RISC complex and thus increasing protein levels. *PVT1*-protein interactions stabilize proteins and prevent their degradation. Pictograms in purple and green illustrate *PVT1*-miRNAs/proteins interactions identified in colorectal and gastric cancer, respectively.

**Figure 6:**
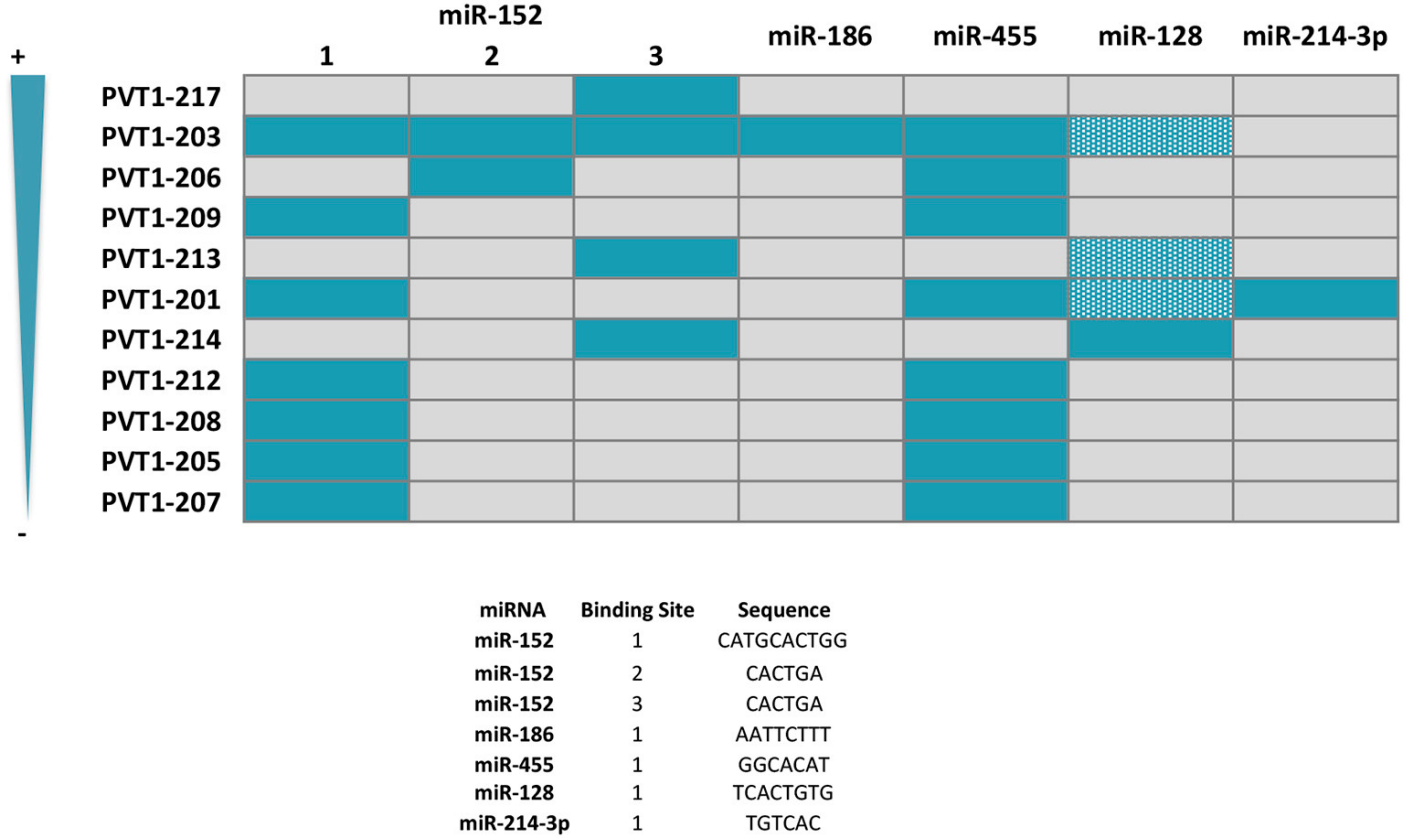
Predicted sponge capacity of miRNAs by *PVT1* transcript variants. *PTV1* isoforms are ranked according to their relative expression abundance (%) in gastrointestinal normal tissues and tumors. Gray and solid blue boxes indicate absence and presence of the nucleotide sequence complementary to the different miRNA, respectively. Patterned blue boxes indicate complementarity with miR-128 principal binding motif, but lack of the additional contact sites and/or difference in length from the main interacting core. Nucleotide sequence of the miRNA binding site is specified.

miR-152 suppresses the proliferation and motility of gastric cancer cells targeting CD151 and FGF2, which are cell surface receptors well-known to participate in the spreading, migration and invasion of tumors (118–120) (Figure 5). miR-152 expression is downregulated in gastric cancer tissues, most likely as a result of the interaction with *PVT1* which has been shown *in vitro* to effectively sponge this miRNA (60, 118). In good agreement, *PVT1* is negatively correlated with miR-152 expression in tumors from gastric cancer patients, and its genetic manipulation in SGC7901 and BGC823 gastric cell lines influences miR-152 levels and ultimately, CD151 and FGF2 expression (60). It is noteworthy that three independent binding sites for miR-152 have been identified in *PVT1*, however not all of them are present in the transcripts more frequently expressed in the gastric tissue (Figure 6). No reports are found in the literature regarding the role of *PVT1* in sponging miR-152 in colorectal tumors. However, as observed for gastric tumorigenesis, this miRNA acts as a tumor suppressor in colorectal cancer cell lines and its expression is downregulated in primary colorectal tumors (121, 122).

#### miR-186

This tumor suppressor miRNA negatively regulates the proliferation, invasion and migration of gastric cancer cells by impairing the EMT process through TWIST1 targeting and degradation (123). miR-186 regulates the expression of hypoxia inducible factor 1α (HIF-1α) and blocks aerobic glycolysis in gastric cancer cell lines (124). HIF-1α is a key protein regulating the response of cells to low levels of oxygen (125). In cancer, HIF-1α is significantly associated with metastasis, stemness and poor prognosis and thus, tumors engage sophisticated mechanisms to keep its expression at high levels (126). Gastric cancer cell lines with *PVT1* overexpression show increased HIF-1α mRNA and protein levels, that are reduced with a miR-186 mimic. Conversely, the decreased HIF-1α levels achieved by *PVT1* downregulation are rescued upon usage of a miR-186 inhibitor (124) (Figure 5). *PVT1* binding to miR-186 occurs through a single interacting site, which according to *in silico* analysis is present exclusively in *PVT1*-Sv203 (Figure 6). miR-186 role in colorectal cancer pathogenesis is not so clear. Some reports have identified miR-186 [5p] as a tumor suppressor due to its ability to inhibit proliferation, metastasis and EMT of colorectal cancer cells by targeting ZEB1, while others point out in the opposite direction (127–129).

#### miR-455

*PVT1* acts as a ceRNA to negatively regulate the expression of miR-455 in HT29 and SW480 colon cancer cell lines (72). Hsa-miR-455 encodes for two miRNAs: miR-455-5p and miR-455-3p. *PVT1* seems to specifically bind to miR-455-5p (Figure 6). This miRNA is frequently downregulated in colorectal tumors and acts predominantly as a tumor suppressor element by targeting phosphoinositide-3-kinase regulatory subunit 1 (PIK3R1) and Runt-related transcription factor 2 (RUNX2), both crucial for the development of tumors (72, 130, 131) (Figure 5). An equivalent tumor suppressor role has been attributed to miR-455 in gastric tumorigenesis (132, 133).

#### miR-128

*In silico* analysis identified *PVT1* Sv-214 as ceRNA for miR-128, and the initial observation was confirmed experimentally in colorectal cancer cell lines through gain and loss-of-function experiments against *PVT1* Sv-214 isoform and further extended to gastric cancer cell line model (68, 79). Moreover, the expression of miR-128 showed a significant inverse correlation with the expression of *PVT1* Sv-214 in colorectal and gastric primary tumor samples. As depicted in Figure 6, the central interacting motif between *PVT1* Sv-214 and miR-128 is present in several transcript variants. However, the additional contact sites between the lncRNA and the miRNA pointed out by the authors are not always present in these variants or alternatively differ in length from the central motif. Therefore, the capacity of these *PVT1* isoforms to sponge miR-128 should be confirmed experimentally. miR-128 targets the Lin28 transcript for degradation in colorectal cancer cell lines (68). Lin28 is an RNA-binding protein that inhibits the processing of let-7 family of miRNAs. Let-7 regulates the translation of mRNAs important for the embryo development, cell pluripotency, metabolism, and tumor progression (68, 134). In line with this observation, the expression of *PTV1* Sv-214 and let7a negatively correlated in colorectal cancer patient samples (Figure 5). Notably, Lin28 cooperates with Wnt signaling and Snail to drive growth and invasiveness in colorectal and gastric tumors, respectively (135–137). Conversely, in gastric cancer cell line systems, miR-128 was shown to target TrkC (79) (Figure 5). TrkC/NTRK3 is a member of the NTRK neurotrophin tyrosine kinase receptor family and functions as a tumor suppressor. TrkC is considered a dependence receptor, which is characterized by its ability to induce opposing biological effects depending on the availability of the ligand, NT-3. In the presence of the TrkC ligand, a survival signal is transduced, whereas its absence results in cleavage of a death-domain peptide and induction of apoptosis. NT-3 levels are reduced in CRC and consistently with the tumor suppressor activity of the receptor, tumors tend to accumulate inactivating point mutations or silence protein expression by means of promoter methylation (138, 139). Interestingly, gastric tumors seem to achieve TrkC inactivation through miR128-mediated transcript degradation.

#### miR-214

Bioinformatics analysis also allowed the identification of *PVT1* as a sponge for miR-214-3p. This miRNA negatively regulates the expression of IRS1 (Insulin receptor substrate 1) in the colon cancer cell line HCT116 (107). IRS1 is a key signaling mediator of the insulin/insulin-like growth factor (IGF) system controlling cellular proliferation, differentiation, EMT, and apoptosis in colorectal cancer (140, 141). Upon phosphorylation by the insulin receptor, IRS1 binds specifically to cellular proteins containing SH2 domains such as phosphatidylinositol 3-kinase (PI3K) regulatory subunit (p85) or GRB2, leading to activation of PI3K and MAPK signaling pathways, respectively (142, 143) (Figure 5). Accordingly, both *PVT1* and miR-214-3p sustained the expression and activation of PI3K catalytic subunit (p110) and Akt in colon cancer cell line models (107). No reports have described so far *PVT1* binding to miR-214 in gastric cancer. However, this might not contribute for stomach carcinogenesis, as miR-214 displays an oncogenic role rather than tumor suppressor in gastric epithelial tumor cells. Specifically, miR-214 was been found upregulated in gastric tumors and sustains cell proliferation, migration and invasion by targeting GSK3β, PTEN, PRDM16, and A2AR (144, 145).

### Protein Stabilization

LncRNA can also interact with proteins and regulate their stability by preventing post-translational modifications associated with protein degradation. Specifically, *PVT1* has been shown to bind and stabilize some proteins that are relevant in the gastrointestinal tumorigenesis, such as STAT3 and Lin28.

#### STAT3

*PVT1* has been shown to interact with transcription factor STAT3 in gastric cancer cell lines. Binding of *PVT1* to STAT3 restricted STAT3 ubiquitin-proteasomal degradation leading to sustained phosphorylation and nuclear translocation of the transcription factor (77) (Figure 5). As described before, STAT3 has a strong role supporting tumor progression and therefore, significant efforts have been aimed at the development of specific and non-toxic inhibitors for cancer treatment (146, 147).

#### LIN28

RNA immunoprecipitation followed by mass spectrometry identified Lin28 as a protein interacting directly with *PVT1* Sv-214 (68). Lin28-*PVT1* complex formation prevented Lin28 degradation by the proteasome, increasing the steady-state levels of this RNA-binding protein. Consistently, Lin28 protein levels were reduced *in vitro* and *in vivo* when *PVT1* was knocked down using siRNAs technology, and increased when *PVT1* was overexpressed (Figure 5). The interaction between Lin28 and *PVT1* is known to take place in the cytoplasm and to involve the 3′ region of *PVT1* Sv-214. Although the specific binding site has not been mapped, it is known to be contained within nucleotides ranging from position 672 and 922 in this *PVT1* isoform and encoded by a small portion of exon 17 and full exons 18 and 19. Therefore, besides *PVT1* Sv-214, only *PVT1* Sv-217 could potentially interact with Lin28. Altogether, Lin28 is regulated by *PVT1* through two independent mechanisms: (1) post-transcriptionally, preventing Lin28 transcript targeting by mir-128, as described above, and; (2) post-translationally, reducing Lin28 protein degradation by the proteasome machinery (68).

### Epigenetic Regulation

**Figure 7:**
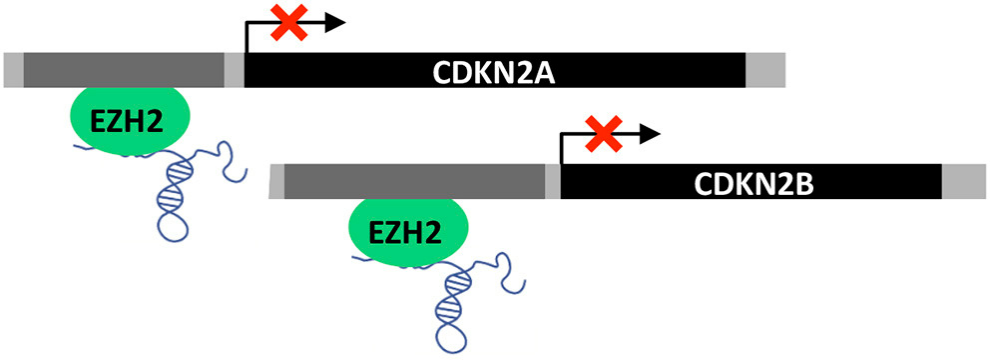
Epigenetic regulation by *PVT1*. *PVT1* recruits EZH2 polycomb group protein leading to promoter methylation and transcriptional repression of *CDKN2A* (p16) and *CDKN2B* (p15).

LncRNAs are important epigenetic regulators and in turn, perturbations of epigenetic regulation are thought to be a key feature of many cancers and even driver events (148). Twenty percent of all human lncRNAs have been shown to physically associate with Polycomb Repressive Complex 2 (PRC2 complex), which operates as a transcriptional repressor (149, 150). *PVT1* has been shown to recruit Enhancer of Zeste homolog 2 (EZH2) to epigenetically negatively regulate the expression of p15 and p16 in gastric cancer cell lines (78). Interestingly, in primary gastric tumors *PVT1* correlates positively with EZH2 and negatively with p15/p16 protein levels (78) (Figure 7).

## *PVT1* as a Prognostic Biomarker

*PVT1* is clearly dysregulated in gastric and colorectal cancer, and experimental mechanistic studies convincingly demonstrate that elevated levels of this lncRNA promote proliferation, angiogenesis and metastasis in human malignancies. Consistently, possible associations between *PVT1* levels and patient prognosis have been investigated by several independent groups. However, as mentioned before, *PVT1* exhibits a significant variability in transcript expression and this has not been rigorously considered when interrogating *PVT1* levels in primary tumors. Most of the studies have determined *PVT1* expression by means of qPCR-based techniques, and surprisingly none of them directly assessed the expression of *PVT1* Sv-217, which according to datasets available in public repositories such as TCGA, is the isoform predominantly expressed in colorectal and stomach tumors (Figure 1) (151). Additionally, primers targeting multiple isoforms have been used in most of the studies, which might account for the differences observed in the clinicopathological features correlating with *PVT1* expression.

In colorectal cancer, the expression of *PVT1* isoform Sv-214 was found to be positively associated with tumor size, advanced stage (III-IV), distant metastasis, and reduced overall and disease-free survival (70). *PVT1* Sv-203 when amplified with qPCR primers that could co-amplify Sv-202 and Sv-211 (66) (two isoforms that according to genome-wide RNAseq profiling have no detectable expression in colorectal malignancies) correlates with advanced stage (III-IV), lymph node metastasis, venous invasion, and decreased overall survival (66, 70). However, the expression of the same *PVT1* isoform Sv-203, when determined together with the expression of Sv-206 and Sv-212 (two isoforms that are moderately expressed in colorectal tumors), was not associated with the clinicopathological features described above (69). Additional studies assessing *PVT1* overall levels by means of high-throughput technologies such as microarrays or RNA sequencing, observed that high expression values were associated with shorter patient overall survival (67, 152). Similarly, in gastric cancer, elevated *PVT1* expression was found to be associated with high tumor stage (III–IV) (75–78), lymph node metastasis (76) and overall/disease-free survival (78). *PVT1* Sv-214 specific evaluation in gastric cancer patients was associated with larger tumor size (5 cm cut off), advanced stage (III-IV) and reduced overall survival (79). Collectively, the available literature supports the contention that elevated expression of *PVT1* is associated with poor prognosis of gastrointestinal cancer patients, but additional isoform-specific studies are warranted.

## *PVT1* and Resistance to Therapeutic Agents

Current treatment for locally advanced colorectal and stomach cancer involves surgical resection of the primary tumor followed by adjuvant chemotherapy to lower the risk of tumor recurrence. Certain patients are also eligible for preoperative or neoadjuvant therapy, being radiation-based treatment the best option for rectal cancer. Several drugs are approved for the treatment of gastrointestinal cancer, but nowadays ECF chemotherapy (epirubicin, cisplatin, and 5-fluorouracil) and FOLFOX/FOLFIRI/FOLFOXIRI regimens (5-fluorouracil, leucovorin, oxaliplatin, and/or irinotecan) constitute the standard of care for stomach and colorectal cancer, respectively (12–15). Intrinsic and acquired resistance to therapeutic agents is the first cause of treatment failure in cancer patients (153). Therefore, identification of the molecular pathways driving the resistance to chemotherapeutic drugs and the screening of drug response biomarkers in cancer patients is of paramount importance to maximize response rates (154–156).

Several studies have investigated the expression of *PVT1* in colorectal and gastric cancer patients treated with different regimens. *PVT1* was found upregulated using quantitative RT-PCR in colorectal cancer patients refractory to 5-fluorouracil (5-FU) in neoadyuvancy (primary resistance), and also in colorectal and gastric cancer patients receiving but not benefiting from cisplatin (70, 108, 157). These findings were consistent with the results obtained using cellular models of acquired drug resistance *in vitro*. HCT116 colon cancer cells resistant to the combination of 5-FU and radiation displayed increased levels of *PVT1* compared to the parental cell line when evaluated using a human lncRNA Expression Array (158). Assessment of *PVT1* transcript levels by quantitative RT-PCR in LOVO and RKO colon cancer cell lines with acquired resistance to cisplatin, or HCT8 and HCT116 with secondary resistance to 5-FU also evidenced the transcriptional upregulation of *PVT1* lncRNA (70, 157). Likewise, BGC823 and SGC7901 gastric cancer cell line models resistant to cisplatin exposure, exhibited increased levels of *PVT1* (108). Of note, all the cell lines used in these studies were all *MYC* amplified, and in the case of the colorectal cancer cell lines, also *PVT1* amplified. It might have been very interesting to investigate to what extent elevated *PVT1* levels by copy-number gain, are required for drug resistance. Interference of *PVT1* expression using siRNA technology in cisplatin resistant cellular models resulted in a reduction of cell proliferation and engagement of apoptosis (70, 108, 157). Mechanistically, some of these effects could be attributed to MDR1 (Multidrug resistance protein 1B) and MRP1 (Multidrug resistance-associated protein 1), which have been shown *in vitro* to modify their expression at mRNA and protein level in a *PTV1*-dependent manner (70, 108, 157). Both proteins are plasma membrane efflux pumps controlling the intracellular bioavailability of substrates at the expense of ATP hydrolysis, and are well-known chemotherapy-resistance mediators in cancer (159).

## Conclusions and Future Perspectives

*PVT1* is an oncogenic lncRNA in many cancer types, including colorectal and stomach cancer. This lncRNA is involved in multifaceted aspects of cancer biology such tumor growth, metastasis, and response to therapeutic agents through a complex signaling network that involves interactions with DNA, RNA, and proteins. *PVT1* complexity starts at the transcriptional level. Twenty-five splice variants have been described for *PVT1* according the latest release of Ensembl database, being 11 of them detectable in colorectal and gastric normal/tumor tissues with the current sequencing platforms. Surprisingly, this complexity has been largely neglected when addressing *PVT1* expression different in tissues. This is of special relevance if as postulated by some authors, *PVT1* is intended to be used as a biomarker for diagnosis, personalized therapeutic treatment, or even a new therapeutic target. Currently, there are no reports associating *PVT1* up-regulation with well-established and clinically relevant molecular features of colorectal and gastric tumors such as microsatellite stability (MSI/MSS), CpG island methylator phenotype (CIMP+/CIMP−), consensus molecular subtypes (CMS1, CMS2, CMS3, and CMS4 for colorectal cancer, and MSI, EBV, CIN, GS for gastric cancer) or mutations in key driver genes (*APC, CTNNB1, SMAD4, TGFB1, TP53, KRAS, PI3K, CDKN2A, CDH1, ERBB2*, or *RHOA*).

Experimentally, more efforts should be invested in the study of the primary, secondary, and higher-order structure of *PVT1* transcripts and *PVT1* promoter region, which is crucial for understanding the function of this lncRNA at a molecular level. The role of *PVT1* in colorectal and gastric cancer has been addressed using gain-and-loss of function experiments using gene overexpression and RNA interference approaches in cell line systems, respectively. *PVT1* encodes multiple miRNAs in intronic regions. The nuclear localization of spliced intronic sequences prevents optimal targeting by the RNAi machinery, which is mainly localized to the cytoplasm. Alternative oligo-mediated RNA knockdown strategies, such as modified antisense oligonucleotides or gapmers, should be used to block *PVT1* miRNA-mediated effects. Ideally, knock-out/knock-in mouse models should be used to better assess the role of *PVT1* in cancer biology. However, compared to protein coding genes, very few animal models have been generated to assess the function of lncRNAs *in vivo*. Unfortunately, many lncRNAs are not highly conserved hindering the identification of mouse orthologs and full function evaluation still represents a challenge.

## Author Contributions

AM-B, DA, and HD wrote the manuscript.

### Conflict of Interest

The authors declare that the research was conducted in the absence of any commercial or financial relationships that could be construed as a potential conflict of interest.
